# Circadian Gene Variants: Effects in Overweight and Obese Pregnant Women

**DOI:** 10.3390/ijms25073838

**Published:** 2024-03-29

**Authors:** Marica Franzago, Paola Borrelli, Pierluigi Cavallo, Luciano Di Tizio, Diego Gazzolo, Marta Di Nicola, Liborio Stuppia, Ester Vitacolonna

**Affiliations:** 1 Department of Medicine and Aging, School of Medicine, and Health Sciences, “G. D’Annunzio” University, Via dei Vestini, Chieti-Pescara, 66100 Chieti, Italy; marica.franzago@unich.it (M.F.); pierluigi.cavallo@studenti.unich.it (P.C.); diego.gazzolo@unich.it (D.G.); 2 Center for Advanced Studies and Technology (CAST), “G. D’Annunzio” University, Chieti-Pescara, 66100 Chieti, Italy; stuppia@unich.it; 3 Laboratory of Biostatistics, Department of Medical, Oral and Biotechnological Sciences, “G. D’Annunzio” University, Chieti-Pescara, 66100 Chieti, Italy; paola.borrelli@unich.it (P.B.); marta.dinicola@unich.it (M.D.N.); 4 Department of Obstetrics and Gynaecology, SS. Annunziata Hospital, “G. D’Annunzio” University, 66100 Chieti, Italy; lucianoditizio@virgilio.it; 5 Neonatal Intensive Care Unit, “G. D’Annunzio” University, 66100 Chieti, Italy; 6 Department of Psychological, Health and Territorial Sciences, School of Medicine and Health Sciences, “G. D’Annunzio” University, Chieti-Pescara, 66100 Chieti, Italy

**Keywords:** GDM, Mediterranean diet, *CLOCK*, *BMAL1*, *CD36*, pregnancy, obesity

## Abstract

Obesity and overweight are common and complex conditions influenced by multiple genetic and environmental factors. Several genetic variants located in the genes involved in clock systems and fat taste perception can affect metabolic health. In particular, the polymorphisms in *CLOCK* and *BMAL1* genes were reported to be significantly related to cardiovascular disease, metabolic syndrome, sleep reduction, and evening preference. Moreover, genetic variants in the *CD36* gene have been shown to be involved in lipid metabolism, regulation of fat intake, and body weight regulation. The aim of this study is to evaluate, for the first time, the association between variants in some candidate genes (namely, *BMAL1* rs7950226 (*G*>*A*), *CLOCK* rs1801260 (*A*>*G*), *CLOCK* rs4864548 (*G*>*A*), *CLOCK* rs3736544 (*G*>*A*), *CD36* rs1984112 (*A*>*G*), *CD36* rs1761667 (*G*>*A*)) and overweight/obesity (OB) in pregnant women. A total of 163 normal-weight (NW) and 128 OB participants were included. A significant correlation was observed between *A*-allele in *CLOCK* rs4864548 and an increased risk of obesity (OR: 1.97; 95% CI 1.22–3.10, *p* = 0.005). In addition, we found that subjects carrying the haplotype of rs1801260-*A*, rs4864548-*A*, and rs3736544-*G* are likely to be overweight or obese (OR 1.47, 95% CI 1.03–2.09, *p* = 0.030), compared with those with other haplotypes. Moreover, a significant relation was observed between third-trimester lipid parameters and genetic variants—namely, *CD36* rs1984112, *CD36* rs1761667, *BMAL1* rs7950226, and *CLOCK* rs1801260. A multivariate logistic regression model revealed that *CLOCK* rs4864548 *A*-allele carriage was a strong risk factor for obesity (OR 2.05, 95% CI 1.07–3.93, *p* = 0.029); on the other hand, greater adherence to Mediterranean diet (OR 0.80, 95% CI 0.65–0.98, *p* = 0.038) and higher HDL levels (OR 0.96, 95% CI 0.94–0.99, *p* = 0.021) were related to a reduced risk of obesity. Interestingly, an association between maternal *CLOCK* rs4864548 and neonatal birthweight was detected (*p* = 0.025). These data suggest a potential role of the polymorphisms in clock systems and in fat taste perception in both susceptibility to overweight/obesity and influencing the related metabolic traits in pregnant women.

## 1. Introduction

Overweight and obesity represent a significant public health issue related to an increased risk of chronic noncommunicable diseases (such as cardiovascular disease and diabetes mellitus). Both overweight and obesity are also the most common clinical conditions in women of reproductive age, representing relevant clinical entities that complicate pregnancies [1], and are known to be caused by gene–environment interactions. These conditions are linked with short- and long-term consequences for the mother [including increasing risk of gestational diabetes mellitus (GDM), hypertension, and preeclampsia] and their offspring [miscarriage, stillbirth, congenital anomalies, shoulder dystocia, macrosomia, and neonatal death] [2,3]. Metabolically, women with obesity present increased insulin resistance from the beginning of pregnancy, which may manifest during pregnancy as glucose intolerance and possible fetal overgrowth [1]. Synchronized circadian rhythms, which are closely related to health [4], consist of approximately 24 h intrinsic oscillations and are affected by the central/primary clock (located in the suprachiasmatic nucleus of the hypothalamus, SCN) and the periphery clock (located in the peripheral tissues) [5,6]. Circadian clocks are molecular machines that organize daily timing, orchestrating a wide range of molecular, physiological, and behavioral processes [7,8]. The molecular machinery of the circadian rhythm is made up of four main proteins, each of which regulates the expression of other downstream genes in a negative feedback loop. In detail, the core of this machinery is BMAL1 (Brain and Muscle ARNT-Like 1)–CLOCK (circadian locomotor output cycles kaput) heterodimer that induces the expression of PER and CRY. These transcription factors, in turn, inhibit the expression of BMAL1 and CLOCK. Thus, the lack of BMAL1 and CLOCK inhibits the expression of CRY and PER so that the cycle can begin again [9]. Pathological changes associated with the circadian gene have been linked to various phenotypes originating from disrupted physiological processes, including cancer and different types of tumors [10,11,12], metabolic diseases [13,14], cardiovascular diseases [15], sleep disorders [16], hypercholesterolemia, and hyperglycemia [17]. Moreover, several studies showed that dysfunction of the circadian clock may be a critical parameter contributing to the development of obesity [18]. Circadian gene variants are reported to be associated with BMI, increasing the risk of overweight and obesity by up to 1.5-fold [19,20,21,22,23]. Nevertheless, few studies have evaluated the relationship between circadian rhythms and reproduction, and circadian rhythm disruption has received less attention in pregnant women. Interestingly, there is growing interest in the role of circadian rhythms in metabolic regulation, as well as the lack of any studies on the role of clock genes and circadian rhythms in pregnant women. Thus, the investigated SNPs in the genes involved in clock systems (*CLOCK* and *BMAL1*) and taste genes (*CD36*) were selected in this study based on their genomic position and their function, according to several genome-wide association studies (GWAS) and the NCBI dbSNP database. In particular, rs7950226 (*G*>*A*) in the *BMAL1* gene and rs1801260 (*A*>*G*), rs4864548 (*G*>*A*), and rs3736544 (*G*>*A*) in the *CLOCK* gene were reported to be significantly correlated with cardiovascular disease, obesity, metabolic syndrome, sleep reduction, and evening preference [22,23,24,25]. Moreover, rs1984112 (*A*>*G*) and rs1761667 (*G*>*A*) in the *CD36* gene have been identified to be involved in lipid metabolism, regulation of fat intake, and body weight regulation [26,27,28,29].

Therefore, the aim of the present study is to investigate the association between variants in candidate genes—namely, *BMAL1* rs7950226 (*G*>*A*), *CLOCK* rs1801260 (*A*>*G*), *CLOCK* rs4864548 (*G*>*A*), and *CLOCK* rs3736544 (*G*>*A*)—and overweight/obesity in 291 pregnant women. The genetic variants of the *CD36* gene, reducing CD36 transcript, could explain the differences in fat perception and fat preferences [26,30]; on the other hand, the relationship between overweight/obesity and SNPs in *CD36* among pregnant women has not been fully clarified. Therefore, two selected variants in *CD36* (rs1984112 *A*>*G* and rs1761667 *G*>*A*) were also included in this study.

## 2. Results

### 2.1. Clinical and Anthropometric Data

The demographic, anthropometric, and clinical parameters of the two groups are presented in Table 1. A total of 291 pregnant women were included in the study: specifically, 163 NW (pre-pregnancy BMI = 21.4 ± 2.0) and 128 OB participants (pre-pregnancy BMI = 32.3 ± 6.4). The mean age of the women was 33.9 ± 5.1 years. There were statistical differences between the two groups regarding age and school education. In particular, the OB women showed lower mean age (33.2 ± 5.3 vs. 34.4 ± 5.0, *p* = 0.05) and lower educational status (*p* < 0.001) compared with controls. In addition, the OB group showed a higher BMI at the end of pregnancy (35.2 ± 6.6 vs. 25.9 ± 2.6, *p* < 0.0001) and a lower increase in average weight gain at delivery vs. the weight recorded before pregnancy than the women of the control group (*p* < 0.001). They also showed higher systolic (120.3 ± 12.8 vs. 113.5 ± 12.5, *p* < 0.001) and diastolic blood pressure (73.4 ± 9.5 vs. 69.2 ± 9.1, *p* = 0.001) when compared with controls. The cases group showed lower serum concentrations of HDL-c (62.0 ± 14.2 vs. 67.2 ± 15.2, *p* = 0.008) during the third trimester than the control group. A significant difference was detected between the two groups regarding the delivery—specifically, the cesarean sections in OB women were higher than in controls (*p* = 0.019). All participants underwent OGTT, and mean glucose values are presented in Table 1.

The percentage of women in the two groups who had a diagnosis of GDM is summarized in Table 1. The OB group showed a higher occurrence of GDM than controls (OR:2.60; 95% CI 1.57–4.27, *p* < 0.001). All GDM women were informed of their hyperglycemic status, and they received the same nutritional advice in accordance with current recommendations. The NW women with GDM required a significantly lower insulin requirement than cases (*p* < 0.001).

### 2.2. Lifestyle Habits

Table 2 shows circadian attitude, Mediterranean diet adherence, smoking habits, and PA in the third trimester of pregnancy. The scores on the rMEQ showed that the sample comprised 33.8% morning type, 3.4% evening type, and 62.7% intermediate type. The distribution of the MEQ results demonstrated no significant differences between the two groups (Table 2). The median PREDIMED score was 9.1 and 8.4 for NW and OB women, respectively (*p* = 0.007). No differences in the PA between the groups were found. Smoking differed significantly between the cases and the control group (*p* = 0.004). In fact, 44.8% and 52.6% of former smokers and non-smokers were found in the cases group, respectively. Other comparisons between groups were not significant.

### 2.3. Newborn’s Data

The main neonatal anthropometric characteristics are shown in Table 3. Mean gestational age at delivery was statistically significantly lower in the OB group (*p* < 0.001) (Table 3). No other differences were found between the two groups.

### 2.4. Genotype Analysis

The genotype distributions of the polymorphisms tested based on additive, dominant, and recessive inheritance genetic models are shown in Table 4. A significant difference was detected in terms of genotype distribution of rs4864548 (*G*>*A*) in *CLOCK* between the OB group and controls (Table 4). In fact, the *AG* genotype was significantly more frequent among OB women compared with controls (53.9% vs. 36.2%, *p* = 0.009). Furthermore, using a dominant inheritance model ((*AA*+*AG*) vs. (*GG*)), the *A*-allele in *CLOCK* rs4864548 was found to be significantly overrepresented in OB cases compared with the controls (OR: 1.97; 95% CI 1.22–3.10, *p* = 0.005). For the other SNPs, in additive, dominant, and recessive inheritance genetic models, no significant differences in the genotype frequency were detected between the two groups. Furthermore, no significant association was found between the six genetic variants studied and GDM status.

**Figure 1:**
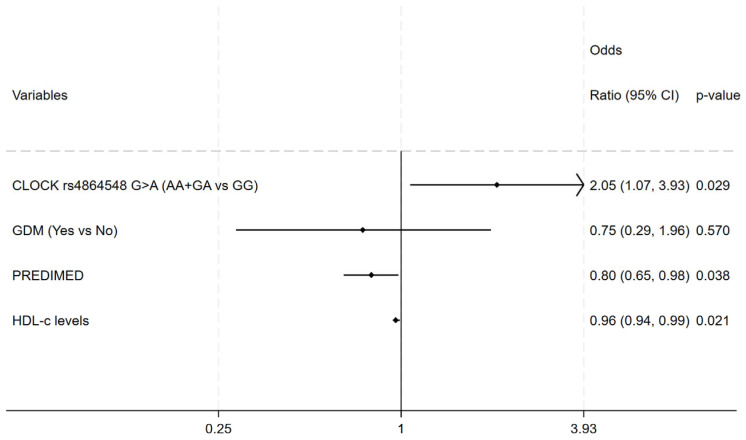
Multivariate logistic regression model for obesity. Independent variables: CLOCK rs4864548, GDM, PREDIMED and HDL-C levels; Adjusted odds ratio (95% confidence interval) and *p*-value.

A significant relation between *CD36* rs1984112 (*A*>*G*) with the third-trimester LDL-C as well as third-trimester TC in OB women was detected. In fact, *G*-allele carriers showed lower serum level of TC and LDL-C compared with *A*-allele carriers in both additive (for TC: *AA* 257.67 ± 52.03; *AG* 252.89 ± 50.48; *GG* 218.56 ± 54.75; *p* = 0.036; for LDL-c: *AA* 149.82 ± 43.23; *AG* 147.63 ± 40.08; *GG* 108.52 ± 46.65; *p* = 0.003) and recessive (for TC: *GG* 218.56 ± 54.76; *AA*+*AG* 254.59 ± 50.51; *p* = 0.011; for LDL-c: *GG* 108.52 ± 46.65; *AA*+*AG* 148.44 ± 41.03; *p* < 0.0001) genetic models. A significant relation was observed between *CD36* rs1761667 (*G*>*A*) and third-trimester LDL-c as well as TC levels in both OB cases (for TC: *GG* 231.74 ± 49.93; *AA*+*GA* 256.43 ± 52.27, *p* = 0.028; for LDL-c: *GG* 126.45 ± 43.47; *AA*+*GA* 149.06 ± 43.07; *p* = 0.017) and controls (for TC: *GG* 229.00 ± 44.44; *AA*+*GA* 254.15 ± 50.11; *p* = 0.023; for LDL-c: *GG* 125.13 ± 43.42; *AA*+*GA* 146.18 ± 41.60; *p* = 0.032; for HDL-c: *GG* 61.08 ± 13.38; *AA*+*GA* 68.67 ± 15.26; *p* = 0.024). Then, an association was observed between *BMAL1* rs7950226 (*G*>*A*) with third-trimester HDL-c levels of NW women in both additive (*AA* 72.77 ± 15.61; *AG* 64.31 ± 15.23; *GG* 66.90 ± 13.92; *p* = 0.047) and recessive (*AA* 72.76 ± 15.61; *AA*+*GA* 65.41 ± 14.67; *p* = 0.019) genetic models. Finally, a significant relation between *CLOCK* rs1801260 (*A*>*G*) and third-trimester TG levels (*AA* 195.31 ± 65.73, *GG*+*AG* 235.84 ± 101.22; *p* = 0.010) was found in the controls. In fact, the *AA* genotype showed lower TG levels compared with *G*-allele carriers in the dominant model. All the investigated genotype frequencies were within the Hardy–Weinberg equilibrium in both the cases and controls. In addition, we found that subjects carrying the haplotype of rs1801260-*A*, rs4864548-*A*, and rs3736544-*G* were likely to be OB compared with those with other haplotypes (36.3% vs. 27.9% *p* = 0.030; OR 1.47, 95% CI 1.03–2.09) Multivariate logistic regression analysis revealed that *CLOCK* rs4864548 *A*-allele carriage was a strong risk factor for obesity (OR 2.05, 95% CI 1.07–3.93, *p* = 0.029); on the other hand, greater adherence to the Mediterranean diet (OR 0.80, 95% CI 0.65–0.98, *p* = 0.038) and higher HDL levels (OR 0.96, 95% CI 0.94–0.99, *p* = 0.021) were related to a reduced risk of obesity (Figure 1). The Hosmer–Lemeshow test indicated a good fit of the model in describing the data (chi-square = 91.55, *p* = 0.439)

Finally, regarding neonatal outcomes, an association between maternal *CLOCK* rs4864548 and neonatal birthweight was detected in dominant genetic models. In detail, *A*- allele carriers showed lower neonatal birthweight compared with *GG* genotype carriers in the cases group (*AA+AG*: 3171.97 ± 447.44; *GG*: 3386.88 ± 422.64; *p* = 0.025).

## 3. Discussion

Obesity is a complex and multifactorial condition to which both lifestyle habits and genetic susceptibility contribute. The increased prevalence of women with overweight and obesity leads to an increase in pregnancies with obesity and, consequently, a global health burden that potentially has several adverse outcomes. CLOCK is a key component of the mammalian molecular clock within the pacemaker neurons of the SCN and plays an important role in fat and glucose metabolism in peripheral organs (including adipose tissue, muscle, and liver) [31,32]. Since circadian disruptions may contribute to different metabolic-related traits, several studies have evaluated the role of common SNPs located in genes involved in clock systems [18]. Previously, Deng et al. [33] identified, through a whole-genome scan and by comparison with earlier studies, the region 4q12, the chromosome location of the *CLOCK* gene, potentially important for obesity phenotypes. In addition, cross-sectional studies have shown associations between this locus and obesity, plasma glucose, hypertension, and T2D, also suggesting its role in cardiovascular risk. Given the emerging evidence showing that alteration in circadian rhythmicity can induce pathophysiological changes, in the present work, we evaluated for the first time the association between overweight/obesity in pregnant women and genetic variants involved with circadian regulation and fat preferences. In this study, we have shown that the *A*-allele in *CLOCK* rs4864548 is found to be significantly more frequent among OB women compared with the controls. In addition, subjects carrying the rs1801260-*A*/rs4864548-*A*/rs3736544-*G* haplotype are likely to be obese compared with those with other haplotypes. Moreover, our results confirm that SNPs in genes involved in the circadian clock and fat taste perception have been linked to third-trimester lipid profiles. As extensively reported in the literature, lipid absorption and metabolism are regulated by some of the core circadian rhythm genes [34,35,36,37,38]. Moreover, the possible interaction between the *CD36* polymorphisms and lipid metabolism has been demonstrated [39]. CD36 receptor is attended in many biological processes, such as oral fat perception, inflammation, angiogenesis, atherosclerosis, immune regulation, and insulin resistance [40,41,42]. Genome-wide linkage scans have identified nearby regions of chromosome 7, where the *CD36* gene is located, associated with TG, HDL-C, and TG/HDL ratio [43]. Therefore, genetic variants in fat taste preference are associated with cardiovascular risk factors, modulating lipid metabolism. In addition, CD36 contributes preferentially to the intake of some nutrients and adversely affects the consumption of others, suggesting interindividual variability in body weight regulation [26]. Future investigations must be conducted to shed light on the functional role of *CD36*, *BMAL1*, and *CLOCK* on lipid metabolism in pregnancy. Obesity prevention, particularly in pregnancy, which is a crucial stage in a woman’s life, has generally focused on modifiable risk factors. The literature supports the importance of lifestyle and dietary habits during pregnancy; in particular, the Mediterranean diet is positively related to maternal and neonatal outcomes [44,45,46,47,48]. In fact, it has been demonstrated that Mediterranean diet adherence is crucial for a correct gestational period with a protective effect on preterm delivery and glucose regulation [49]. In this view, our multivariate logistic regression model showed that *CLOCK* rs4864548 *A*-allele carriage was a strong risk factor for obesity. On the other hand, in agreement with the literature, the obesity risk decreased in pregnant women with higher HDL levels and higher adherence scores to the Mediterranean diet. These data strengthen the important role of modifiable risk factors, specifically Mediterranean dietary patterns, and suggest that targeted health and lifestyle interventions could attenuate genetic susceptibility. Therefore, lifestyle modifications, such as a higher Mediterranean diet adherence or smoking cessation, as well as physical activity, represent a promising strategy for reducing the risk of other complications in pregnancy and further in time. Pregnancy could be affected by epigenetic and environmental factors contributing to the complexity of the metabolic status of both mothers and their offspring [50]. An association between maternal *CLOCK* rs4864548 and neonatal birthweight was detected in this study. It is possible to assume that some effects of the regulatory genetic variants on body weight may be due to their effects on their expression level. Therefore, an interaction between *CLOCK* gene variants, which are located in regulatory sites, and lifestyle habits could also be possible; whereby, DNA methylation of this gene could induce the silencing of their expression and, eventually, consequences for the child and adult health. In conclusion, our study, for the first time, shows an association between *CLOCK* genetic variants and obesity in pregnant women, also strengthening the link between the rs1801260*A*/rs4864548-*A*/rs3736544-*G* haplotype of the core clock gene and the susceptibility to obesity. The present study has some limitations. Epigenetic alterations should be explored as potential molecular mechanisms of the effects occurring during pregnancy, and additional research identifying other plausible pathways is needed. Given the potential role of the genotype of *CLOCK* on gene expression, it is necessary to analyze the effect of genotypes on epigenetic mechanisms, particularly on DNA methylation levels. Future studies will be needed to understand the gene–diet interactions and how circadian disturbances during pregnancy may influence the development of chronic diseases, including diabetes, cardiovascular disease, and cancer, in both mothers and offspring.

## 4. Materials and Methods

### 4.1. Study Design and Participants

A total of 291 Caucasian pregnant women were recruited at the Diabetes, Nutrition, and Metabolism Unit and/or the Obstetrics and Gynaecology Clinic at the Hospital of University “Gabriele d’Annunzio” in Chieti-Pescara. This cross-sectional study received approval from the Ethics Committee of the Province di Chieti and Pescara (protocol code: richxrked; date of approval: 12 December 2019) in accordance with the Helsinki Declaration. All participants signed and gave back written informed consent prior to their participation in the study. The inclusion criteria consisted of pregnant women 18 years of age or older. The exclusion criteria: women suffering from pre-pregnancy diabetes, overt diabetes, or monogenic diabetes, and women with other chronic diseases. Overweight and obese (OB) pregnant women (cases) had pre-pregnancy body mass index (BMI) ≥ 25 and ≥30 kg/m^2^, respectively. Normal weight (NW) pregnant women (controls) were included if they reported pre-pregnancy body mass index (BMI) ≥ 18.5 and <25 kg/m^2^.

### 4.2. Anthropometric and Clinical Measurements

At the first medical examination, sociodemographic characteristics as well as clinical and anthropometric parameters were collected, including pre-pregnancy BMI and blood pressure.

In the third trimester of pregnancy, lipid profile and fasting plasma glucose were collected. At the end of pregnancy, BMI at the end of the pregnancy and gestational weight gain were recorded. Women’s body weight and height were measured using mechanical scales with a stadiometer while participants wore light clothing and no shoes. BMI was calculated by applying the following formula: weight in kilograms/(height in meters × height in meters). Gestational diabetes mellitus (GDM) diagnosis was proved both at the 16–18th and/or at the 24–28th weeks of gestation according to the International Association of Diabetes and Pregnancy Study Groups (IADPSG) criteria [51]. In accordance with current guidelines, a blood glucose test was performed by the glucose reductase method, and HbA1c was measured according to the IFCC (International Federation of Clinical Chemistry and Laboratory Medicine) system [52].

In detail, the diagnosis of GDM was made by 75 g OGTT when any of the following plasma glucose levels were met or exceeded: fasting of 5.1 mmol/L (92 mg/dL), 10.0 mmol/L (180 mg/dL) at 1 h, and 8.5 mmol/L (153 mg/dL) at 2 h [51]. Furthermore, the rate of women needing insulin therapy was registered.

### 4.3. Lifestyle Questionnaires

Circadian typology (chronotype), Mediterranean diet adherence, smoking habits, and physical activity of the women were assessed in the third trimester of pregnancy. The interindividual circadian attitude known as chronotype was evaluated using a 5-item version of the Morningness–Eveningness Questionnaire (MEQr). The scale includes 5 questions taken from the original 19-item version of the MEQ, (the most widely used morningness measure) [53]. According to Italian cutoff criteria, MEQr distinguishes three different chronotypes defined as follows: (i) “morning” (19–25 score), (ii) “intermediate” (11–18 score), and (iii) “evening” (4–10 score) types [54]. In addition, the International Physical Activity Questionnaire (IPAQ-short version) was used to assess physical activity (PA) level [55] generating three different intensity levels (low, moderate, and high PA). The 14-point Mediterranean Diet Adherence Screener (MEDAS), previously validated in the PREDIMED study [56], was used to assess adherence to the Mediterranean diet (Med-Diet). The questionnaire consists of 14 items related to food commonly consumed in the Mediterranean area, recording the frequency of consumption of the food items in servings per day or week. Each question was scored 0 or 1, and the final PREDIMED score ranged from 0 to 14 [57]. Therefore, a score of less than or equal to 5 points indicates no adherence, 6 to 9 indicates medium adherence, and greater than or equal to 10 points indicates a maximum adherence to the Med-Diet.

### 4.4. Newborn’s Measurements

Clinical information relating to newborns was collected at birth from a review of clinical records. Data included mode of delivery, gestational age, sex, and anthropometric measurements such as weight, height, and head circumference. Apgar scores were recorded.

### 4.5. Genetic Analysis

The genetic analysis was conducted at the Laboratory of Molecular Genetics, School of Medicine and Health Sciences, “G. d’Annunzio” University of Chieti-Pescara. Venous blood from each woman was collected in an EDTA tube and stored in controlled conditions at +4 °C until DNA extraction. Genomic DNA was automatically extracted and purified from peripheral blood lymphocytes, using MagPurix Blood DNA Extraction Kit 200 and MagPurix 12 EVO instrument, according to the manufacturer’s instructions (Zinexts Life Science Corp. (New Taipei City, Taiwan)). Nucleic acids were quantified by measuring UV absorption using the NanoPhotometer NP80 instrument (Implen, Inc., Westlake Village, CA, USA). SNPs genotyping was conducted with real-time PCR and TaqMan chemistry using the QuantStudio 5 real-time instrument, according to the manufacturer’s instructions (Thermo Fisher, Waltham, MA, USA). Briefly, the TaqMan method can exploit multiple channels thanks to the use of fluorescent probes with different labels. The most used fluorochromes are FAM (6-carboxy-fluorescein) and VIC (4′,7-dichloro-5′,7′-dimethoxyl-fluorescein). FAM-VIC combined in a single reaction tube allows for multiple analyses simultaneously, detecting both the wild-type allele and mutant allele. The reaction MIX was prepared by the addition of 12.5 μL TaqPathTM ProAmpTM Master Mix, 1.25 μL TaqMan^®^ SNP Genotyping Assay (20×) (Table 5), genomic DNA up to 11.25 μL, and nuclease-free water to a total volume of 25 μL. The reaction was optimized with 20 ng of starting DNA. PCR conditions were 95 °C for 10 min, 40 cycles of 95 °C for 15 s, and 60 °C for 1 min. After the amplification, the raw data were analyzed with Design & Analysis 2 (DA2) software.

### 4.6. Statistical Analysis

Descriptive analysis was carried out using mean and standard deviation (SD) or median and interquartile range (IQR) for the quantitative variables and percentage values for the qualitative ones. Normality distribution for quantitative variables was assessed by the Shapiro–Wilk test. The association between categorical data was investigated by Pearson χ^2^ or Fisher’s exact test and the Student’s *t*-test for independent data or analysis of variance (ANOVA) for more than two groups of continuous data. In addition, the Bonferroni test was used for multiple comparisons. Crude odds ratio (ORs) and corresponding 95% confidence interval (CI) were calculated in order to quantify the risk associated with obesity. A multivariable logistic regression model was performed to identify the mutually adjusted effect among obesity and the independent variables chosen on the basis of (1) the statistical significance (univariate analysis, *p* ≤  0.05); (2) the clinical judgment and their contribution to the model fit (likelihood-ratio test). The goodness of fit of the multivariable logistic regression model was assessed by the Hosmer–Lemeshow test. For each investigated locus, Hardy–Weinberg equilibrium was calculated (χ^2^ *p* > 0.05). A statistical significance was set at the level of ≤0.05 unless adjustment for multiple comparisons was needed. All analyses were performed using Stata software v18.0 MP (StataCorp, College Station, TX, USA) and haplotype frequencies were estimated by the Arlequin software (version 3.5) [58]. Haplotypes were composed of *CLOCK* variants (rs1801260, rs4864548, and rs3736544).

## Figures and Tables

**Table 1:** Demographic, anthropometric, and clinical data of NW and OB pregnant women expressed as mean and standard deviation (SD), absolute frequency (n), and column percentage (%).

Variable	Total	NW	OB	
	n = 291	n = 163	n = 128	*p*-Value
Age (years), mean (SD)	33.9 (5.1)	34.4 (5.0)	33.2 (5.3)	**0.050**
School education, n (%)				
Low school	29 (10.0%)	9 (5.6%)	20 (15.7%)	**<0.001**
High school	147 (50.9%)	73 (45.1%)	74 (58.3%)	
University degree	113 (39.1%)	80 (49.4%)	33 (26.0%)	
Employment, n (%)				
Employed	232 (80.6%)	123 (76.4%)	109 (85.8%)	**0.045**
Unemployed	56 (19.5%)	38 (23.6%)	18 (14.2%)	
Pre-pregnancy weight (kg), mean (SD)	70.9 (19.7)	57.6 (6.8)	87.7 (17.8)	**<0.001**
Weight at the end of pregnancy (kg), mean (SD)	81.4 (19.0)	69.9 (8.5)	96.2(18.0)	**<0.001**
Pre-pregnancy BMI (kg/m^2^), mean (SD)	26.2 (7.1)	21.4 (2.0)	32.3 (6.4)	**<0.001**
BMI at the end of pregnancy (kg/m^2^), mean (SD)	30.0 (6.5)	25.9 (2.6)	35.4 (6.2)	**<0.001**
Weight variation (kg), mean (SD)	10.8 (6.4)	12.3 (5.0)	8.9 (7.4)	**<0.001**
Systolic blood pressure (mmHg), mean (SD)	116.4 (13.1)	113.3 (12.5)	120.3 (12.8)	**<0.001**
Diastolic blood pressure (mmHg), mean (SD)	71.1 (9.5)	69.2 (9.1)	73.4 (9.5)	**0.001**
Glycated hemoglobin (HbA1c)), mean (SD)	5.2 (0.4)	5.1 (0.4)	5.3 (0.4)	0.066
Third-trimester TC (mg/dL), mean (SD)	249.1 (51.0)	249.2 (49.9)	249.0 (52.6)	0.976
Third-trimester HDL-C (mg/dL), mean (SD)	64.8 (14.9)	67.2 (15.2)	62.0 (14.2)	**0.008**
Third-trimester TG (mg/dL), mean (SD)	223.9 (97.5)	217.7 (89.1)	231.6 (106.9)	0.280
Third-trimester LDL-C (mg/dL), mean (SD)	142.1 (43.2)	142.1 (42.6)	142.1 (44.2)	0.987
OGTT (mg/dL) at baseline (min), mean (SD)	88.1 (12.3)	85.2 (12.2)	91.9 (11.5)	**<0.001**
OGTT (mg/dL) after 60 min, mean (SD)	147.5 (37.9)	140.7 (38.7)	156.2 (35.2)	**<0.001**
OGTT (mg/dL) after 120 min, mean (SD)	122.3 (31.8)	118.4 (33.3)	127.4 (29.2)	**0.018**
Delivery, n (%)				
Vaginal delivery	166 (67.5%)	109 (73.2%)	57 (58.8%)	**0.019**
Cesarean section	80 (32.5%)	40 (26.8%)	40 (41.2%)	
GDM, n (%)				
No	113 (38.8%)	79 (48.5%)	34 (26.6%)	**<0.001**
Yes	178 (61.2%)	84 (51.5%)	94 (73.4%)	
Insulin therapy, n (%)				
No	197 (68.4%)	135 (83.3%)	62 (49.2%)	**<0.001**
Yes	91 (31.6%)	26 (16.7%)	64 (50.8%)	

Statistically significant values are in bold. GDM: gestational diabetes mellitus; TC: total cholesterol; HDL-C: high-density lipoprotein cholesterol; LDL-C: low-density lipoprotein cholesterol; TG: triglycerides; OGTT: oral glucose tolerance test.

**Table 2:** Lifestyle habits for cases and controls.

Variable	Total	NW	OB	
	n = 291	n = 163	n = 128	*p*-Value
Smoking history, n (%)				**0.004**
Non-smoker	165 (61.8%)	104 (68.9%)	61 (52.6%)	
Smoker	11 (4.1%)	8 (5.3%)	3 (2.6%)	
Former smoker	91 (34.1%)	39 (25.8%)	52 (44.8%)	
Predimed, mean (SD)	8.7 (1.7)	9.1 (1.7)	8.4 (1.7)	**0.007**
Predimed class, n (%)				0.182
No adherence	9 (4.4%)	4 (4.0%)	5 (4.8%)	
Medium adherence	124 (60.2%)	55 (54.5%)	69 (65.7%)	
Max adherence	73 (35.4%)	42 (41.6%)	31 (29.5%)	
rMEQ, mean (SD)	16.7 (3.4)	16.8 (3.6)	16.6 (3.2)	0.660
rMEQ class, n (%)				0.397
Morning	69 (33.8%)	36 (35.6%)	33 (32.0%)	
Evening	7 (3.4%)	5 (5.0%)	2 (1.9%)	
Intermediate	128 (62.7%)	60 (59.4%)	68 (66.0%)	
IPAQ, n (%)				0.838
Low	152 (54.9%)	88 (56.1%)	64 (53.3%)	
Moderate	76 (27.4%)	43 (27.4%)	33 (27.5%)	
High	49 (17.7%)	26 (16.6%)	23 (19.2%)	

Statistically significant values are in bold.

**Table 3:** Neonatal outcomes relative to NW and OB women.

Variables	Total * n = 250	NW n = 150	OB n = 100	*p*-Value
Gestational week, mean (SD)	39.0 (1.3)	39.3 (1.3)	38.6 (1.3)	**<0.001**
Gender, n (%)				
Male	119 (47.6%)	77 (51.3%)	42 (42.0%)	0.148
Female	131 (52.4%)	73 (48.7%)	58 (58.0%)	
Birth weight (g), mean (SD)	3232.2 (473.4)	3227.8 (491.3)	3238.8 (447.8)	0.858
One-minute Apgar scores, mean (SD)	8.6 (1.1)	8.7 (1.1)	8.5 (1.1)	0.277
Five-minute Apgar scores, mean (SD)	9.6 (0.9)	9.6 (1.0)	9.6 (0.7)	0.408
Birth head circumference (cm), mean (SD)	34.4 (2.2)	34.7 (2.6)	34.1 (1.7)	0.077
Birth length (cm), mean (SD)	49.8 (2.0)	50.0 (2.0)	49.5 (2.0)	0.069

Statistically significant values are in bold. * The neonatal data were available for a total of 250 women.

**Table 4:** Genotype distributions of the polymorphisms for cases and controls.

Additive Inheritance Model	Total	NW	OB	
	n = 291	n = 163	n = 128	*p*-Value
*CD36* rs1984112 *A*>*G*				
*AA*	104 (35.7%)	63 (38.7%)	41 (32.0%)	0.445
*AG*	147 (50.5%)	80 (49.1%)	67 (52.3%)	
*GG*	40 (13.7%)	20 (12.3%)	20 (15.6%)	
*CD36* rs1761667 *G*>*A*				
*AA*	80 (27.5%)	46 (28.2%)	34 (26.6%)	0.337
*AG*	139 (47.8%)	82 (50.3%)	57 (44.5%)	
*GG*	72 (24.7%)	35 (21.5%)	37 (28.9%)	
*BMAL1* rs7950226 *G*>*A*				
*AA*	65 (22.3%)	41 (25.2%)	24 (18.8%)	0.309
*AG*	137 (47.1%)	71 (43.6%)	66 (51.6%)	
*GG*	89 (30.6%)	51 (31.3%)	38 (29.7%)	
*CLOCK* rs1801260 *A*>*G*				
*AA*	143 (49.1%)	79 (48.5%)	64 (50.0%)	0.447
*AG*	123 (42.3%)	67 (41.1%)	56 (43.8%)	
*GG*	25 (8.6%)	17 (10.4%)	8 (6.2%)	
*CLOCK* rs4864548 *G*>*A*				
*AA*	29 (10.0%)	17 (10.4%)	12 (9.4%)	**0.009**
*AG*	128 (44.0%)	59 (36.2%)	69 (53.9%)	
*GG*	134 (46.0%)	87 (53.4%)	47 (36.7%)	
*CLOCK* rs3736544 *G*>*A*				
*AA*	38 (13.1%)	24 (14.7%)	14 (10.9%)	0.442
*AG*	145 (49.8%)	83 (50.9%)	62 (48.4%)	
*GG*	108 (37.1%)	56 (34.4%)	52 (40.6%)	
Dominant inheritance model				
*CD36* rs1984112 *A*>*G*				
*AA*	104 (35.7%)	63 (38.7%)	41 (32.0%)	0.242
*GG*+*AG*	187 (64.3%)	100 (61.3%)	87 (68.0%)	
*CD36* rs1761667 *G*>*A*				
*GG*	72 (24.7%)	35 (21.5%)	37 (28.9%)	0.145
*AA*+*GA*	219 (75.3%)	128 (78.5%)	91 (71.1%)	
*BMAL1* rs7950226 *G*>*A*				
*GG*	89 (30.6%)	51 (31.3%)	38 (29.7%)	0.769
*AA*+*GA*	202 (69.4%)	112 (68.7%)	90 (70.3%)	
*CLOCK* rs1801260 *A*>*G*				
*AA*	143 (49.1%)	79 (48.5%)	64 (50.0%)	0.795
*GG*+*AG*	148 (50.9%)	84 (51.5%)	64 (50.0%)	
*CLOCK* rs4864548 *G*>*A*				
*GG*	134 (46.0%)	87 (53.4%)	47 (36.7%)	**0.005**
*AA*+*GA*	157 (54.0%)	76 (46.6%)	81 (63.3%)	
*CLOCK* rs3736544 *G*>*A*				
*GG*	108 (37.1%)	56 (34.4%)	52 (40.6%)	0.272
*AA*+*GA*	183 (62.9%)	107 (65.6%)	76 (59.4%)	
Recessive inheritance model				
*CD36* rs1984112 *A*>*G*				
*GG*	40 (13.7%)	20 (12.3%)	20 (15.6%)	0.409
*AA*+*AG*	251 (86.3%)	143 (87.7%)	108 (84.4%)	
*CD36* rs1761667 *G*>*A*				
*AA*	80 (27.5%)	46 (28.2%)	34 (26.6%)	0.753
*GG*+*GA*	211 (72.5%)	117 (71.8%)	94 (73.4%)	
*BMAL1* rs7950226 *G*>*A*				
*AA*	65 (22.3%)	41 (25.2%)	24 (18.8%)	0.193
*GG*+*GA*	226 (77.7%)	122 (74.8%)	104 (81.2%)	
*CLOCK* rs1801260 *A*>*G*				
*GG*	25 (8.6%)	17 (10.4%)	8 (6.2%)	0.207
*AA*+*AG*	266 (91.4%)	146 (89.6%)	120 (93.8%)	
*CLOCK* rs4864548 *G*>*A*				
*AA*	29 (10.0%)	17 (10.4%)	12 (9.4%)	0.766
*GG*+*GA*	262 (90.0%)	146 (89.6%)	116 (90.6%)	
*CLOCK* rs3736544 *G*>*A*				
A*A*	38 (13.1%)	24 (14.7%)	14 (10.9%)	0.341
*GG*+*GA*	253 (86.9%)	139 (85.3%)	114 (89.1%)	

Significant values are given in bold.

**Table 5:** The genetic variants selected in this study and the TaqMan SNP genotyping assay.

NCBI SNP Reference	MB Position	Location	TaqMan SNP Assay
*CD36* rs1984112 (*A*>*G*)	Chr.7: 80613604 on GRCh38	5′ flanking exon 1A	C__12093946_10
*CD36* rs1761667 (*G*>*A*)	Chr.7: 80615623 on GRCh38	5′ flanking exon 1A	C___8314999_10
*CLOCK* rs1801260 (*A*>*G*)	Chr.4: 55435202 on GRCh38	3′UTR	C___8746719_20
*CLOCK* rs4864548 (*G*>*A*)	Chr.4: 55547636 on GRCh38	Upstream	C__11821276_10
*BMAL1* rs7950226 (*G*>*A*)	Chr.11: 13296592 on GRCh38	Intron 1	C__11578388_10
*CLOCK* rs3736544 (*G*>*A*)	Chr.4: 55443825 on GRCh38	Exon 20 (p.ASN588=)	C__22273263_10

NCBI, National Center for Biotechnology Information; MP position, Mapped chromosome position; ASN = asparagine.

## Data Availability

The data that support the findings of this study are available from the corresponding author upon reasonable request.
